# Normal Anatomical Features and Variations of the Vertebrobasilar Circulation and Its Branches: An Analysis with 64-Detector Row CT and 3T MR Angiographies

**DOI:** 10.1155/2013/620162

**Published:** 2013-04-29

**Authors:** Veysel Akgun, Bilal Battal, Yalcin Bozkurt, Oguzhan Oz, Salih Hamcan, Sebahattin Sari, Hakan Akgun

**Affiliations:** ^1^Gulhane Military Medical School, Department of Radiology, Etlik, 06018 Ankara, Turkey; ^2^Gulhane Military Medical School, Department of Neurology, Etlik, 06018 Ankara, Turkey; ^3^Balikesir Military Hospital, Department of Radiology, Merkez, 10030 Balikesir, Turkey

## Abstract

*Purpose*. To determine the normal anatomical features and variations of the vertebrobasilar circulation and its branches in patients who underwent multidetector computed tomography (CT) or magnetic resonance (MR) angiographies of the brain. *Methods*. 135 patients (male, 83 and female, 52; mean age, 50.1 years) who underwent CT (*n* = 71) or MR (*n* = 64) angiographies of the vertebrobasilar vasculature for various reasons were analyzed retrospectively. The right and left distal vertebral arteries (VAs), posterior inferior cerebellar arteries (PICAs), anterior inferior cerebellar arteries (AICAs), superior cerebellar arteries (SCAs), posterior cerebral arteries (PCAs), and posterior communicating arteries (PCoAs) were analyzed individually. *Results*. In 24.4% of the cases (33/135) right PICA, in 19.3% of the cases (26/135) left PICA, in 17.8% of the cases (24/135) right AICA, and in 18.5% of the cases (25/135) left AICA were absent. In cases without PICA or AICA, there was a statistically significant, moderately or well-developed AICA or PICA on the same side, respectively (*P* < 0.001). The most common variation was isolated absence of right PICA and was seen in 17.8% of the cases. *Conclusions*. The anatomic features of the branches of the vertebrobasilar circulation may be different from well-known normal anatomy. CT and MR angiographies allow a precise and detailed evaluation of vertebrobasilar circulation.

## 1. Introduction

The vertebrobasilar system that is also known as a posterior circulation is an important vascular network that supplies blood to the posterior part of the cerebral hemispheres including the occipital lobes and the posterior portions of the temporal lobes, the cerebellum, and the brainstem. This system supplies approximately 20% of the intracranial blood flow [1–3]. 

**Figure 1:**
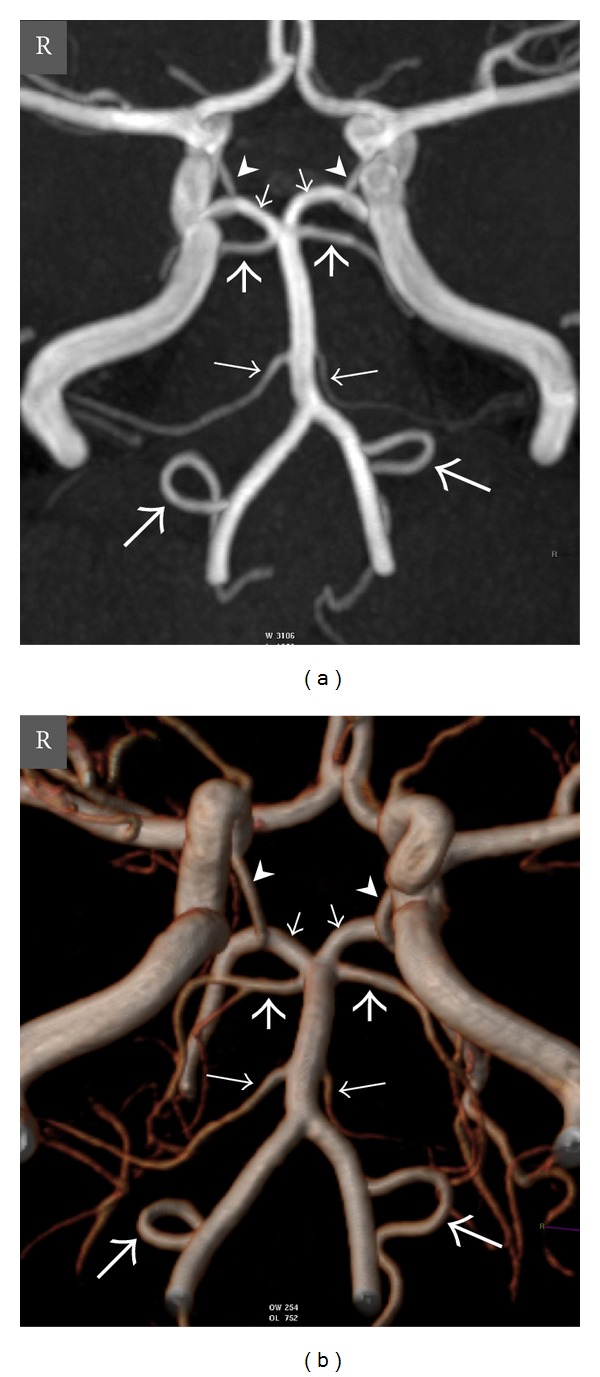
Oblique coronal view maximum intensity projection (MIP) (a) and volume-rendered (VR) (b) time-of-flight (TOF) magnetic resonance (MR) angiography images show normal anatomy of the vertebrobasilar circulation in a 20-year-old woman. *Thick long arrows *= right and left posterior inferior cerebellar arteries (PICAs), *thin long arrows *= right and left anterior inferior cerebellar arteries (AICAs), *thick short arrows *= right and left superior cerebellar arteries (SCAs), *thin short arrows *= right and left posterior cerebral arteries (PCAs), *arrowheads* = right and left posterior communicating arteries (PCoAs), and *R* = right.

According to well-known anatomic configuration the vertebral arteries (VAs) unite to form the basilar artery at the base of the pons. Prior to union, distal vertebral arteries gave rise to posterior inferior cerebellar arteries (PICAs) that supply posteroinferior parts of the cerebellar hemispheres. The basilar artery ascends in a shallow groove on the anterior surface of the Pons. The basilar artery supplies portions of the cerebellum through the anterior inferior cerebellar arteries (AICAs) arising at the junction of its proximal and middle thirds, as well as the superior cerebellar arteries (SCAs) originating just prior to its termination (Figure 1). The basilar artery divides into the 2 posterior cerebral arteries (PCAs) at the level of the proximal midbrain, just after passing the oculomotor nerves. Many studies and case reports demonstrated various anatomic variations of the vertebrobasilar circulation and its branches [1–7]. The anatomical features and variations of the vertebrobasilar system must be well known for accuracy of the interpretation of the ischemic areas, diagnoses, endovascular interventions, and posterior cranial fossa surgeries. 

Although its invasive nature and requirement for ionizing radiation significantly limit its role, digital subtraction angiography (DSA) is regarded as the gold standard technique in the evaluation of vascular structures. With the recent advances in both multidetector computed tomography (CT) technology and high magnetic field magnetic resonance (MR) scanner, even very fine calibrated vascular structures can be depicted easily and as a consequence, the number of the invasive DSA examinations is reduced. Recent studies show that CT angiography is an appropriate method to evaluate the vascular structures [8–10]. With the introduction of the 3T MR equipments, MR angiography, which does not require ionizing radiation and can be performed without contrast medium, has become an advanced and accurate technique in depicting even very fine calibrated intracranial vascular structures [11, 12]. 

The aim of this study was to determine the normal anatomical features and variations of the vertebrobasilar circulation and its branches in patients who underwent CT or MR angiographies of the brain for various reasons.

## 2. Materials and Methods

### 2.1. Patients

Approval for this retrospective study was obtained from the institutional review board. One hundred eighty-eight consecutive patients who underwent CT (*n* = 99) or MR (*n* = 89) angiographies of the vertebrobasilar circulation and its branches for various reasons from February 2011 to November 2011 were analyzed retrospectively. The cases with occlusion, stenosis, or aneurysm in main vertebrobasilar arteries and also the cases with acute or chronic infarcts in areas supplied by the posterior circulation such as cerebellum, brainstem, or occipital lobes (*n* = 31) are excluded. Additionally, 22 patients were excluded due to suboptimal CT angiography (*n* = 13) or MR angiography (*n* = 9) examination due to nonoptimal imaging technique or motion artifacts that were precluding precise analysis of the vertebrobasilar circulation and its branches. The remaining 135 patients were included in this study.

### 2.2. CT Angiography Protocol

CT angiography examinations were performed by using a 64-detector scanner (Aquilion 64, Toshiba Medical Systems, Otawara, Japan). In our standard CT protocol for brain examinations, a scanogram area from the aortic arch to the vertex level in a supine position was adopted as field of view (FOV). During examination, an 18 to 20 gauge angiocath in the antecubital vein was used to inject 70–120 mL of nonionic iodinated contrast media using bolus-tracking method with an automatic injector at a rate of 5 mL/sec and 30 mL of saline solution at a rate of 3 mL/sec (Medrad Stellant D Dual Syringe CT Injection System, Indianola, PA, USA), respectively. The region of interest was positioned at the aortic arch, and the threshold for CT angiography was set as 150 HU. When the threshold was surpassed, helical scanning was automatically initiated. Scan parameters were 120 kV, 300 mA, 400 msec rotation time with a slice thickness of 0.5 mm and increments of 0.4 mm, using a detector collimation of 64 × 0.5 mm (pitch: 0.64). 

### 2.3. MR Angiography Protocol

Subjects were imaged using a 3T MR scanner (Achieva X-Series; Philips Healthcare, Best, the Netherlands) and an 8-channel SENSE receiver head coil in a supine position. Three-dimensional (3D) time of Flight (TOF) MR angiography was obtained in an axial plane with the following parameters: bandwidth, 215.1 Hz; repetition time/echo time (TR/TE), 25/3.5 ms; flip angle, 20°; field-of-view (FOV), 200 × 200 × 100 mm; slab thickness, 25 mm with 4 slabs (200 sections) covering an area from 1st cervical vertebrae to the level of centrum semiovale; matrix size, 496 × 284; slice thickness, 1 mm with 0.5 mm reconstruction; 1 signal averages and acquisition time 8 min 59 sec. Scanned voxel volume was 0.40 × 0.70 × 1.0 mm (0.28 mm^3^); reconstructed voxel volume was 0.36 × 0.36 × 0.50 mm (0.06 mm^3^).

### 2.4. Image Analysis

The obtained axial images from both CT and MR angiographies were transferred to a workstation for analysis. In addition to the axial source data, postprocessed multiplanar reformatted (MPR), maximum-intensity projection (MIP), and 3D volume-rendering (VR) images were evaluated by two radiologists (with 4 and 6 years of experience in CT and MR angiographic imaging), and the decisions were made in consensus. The anatomic features of the right and left distal VAs, PICAs, AICAs, SCAs, PCAs, PCoAs, and basilar artery were analyzed, and anatomical variations were recorded. The presences and origins of all of the branches of the vertebrobasilar system that are listed above were carefully interpreted. The patencies of the PCoAs were also evaluated. The diameters of the distal VAs and the branches of the vertebrobasilar trunk were measured. The diameters of the VAs were measured on axial plane just after entering foramen magnum. The diameters of the branches were measured 0.5 cm distal to their origins. The difference in the diameter of the distal vertebral arteries more than 1 mm was accepted as a criterion for determining the vertebral dominancy. If the difference was smaller than or equal to 1 mm it was accepted as codominancy. 

 The PICAs and AICAs were classified into three groups according to their diameters. For PICA, diameters equal to or over 2 mm, between 1 and 2 mm, and equal to or lower than 1 mm were determined as well, moderate, and poorly developed, respectively. For AICA, diameters equal to or over 1.5 mm, between 0.7 and 1.5 cm, and equal to or lower than 0.7 mm were determined as well, moderately and poorly developed, respectively. For PoCAs the diameters equal to or over 1 mm and below 1 mm were classified into two groups as well and poorly developed, respectively. Fenestrations and hypoplastic segments in a few cases were also noted. 

### 2.5. Statistical Analysis

For discrete and continuous data, percentage (%) and median (min-max) values were used in descriptive statistics, respectively. A Kolmogorov-Smirnov test was used to assess normal distribution. Our study group was not showing normal distribution, and therefore nonparametric tests were used. For comparison between groups with discrete variables and continuous variables, chi-square and Mann-Whitney *U* tests were used, respectively. The changes of the diameters of ipsilateral PICAs and AICAs were evaluated by Spearman's rho. All statistical analyses were made by using SPSS release 15.0 program (SPSS for Windows; SPSS, Chicago, IL, USA). *P* values less than 0.05 were accepted as significant.

## 3. Results

One hundred thirty-five patients (male, 83 and female, 52; mean age, 50.1 years; age range, 10–85 years) were included in this study. Mean diameter of the VAs was 3.08 ± 0.53 mm (right 2.95 ± 0.47, left 3.23 ± 0.57) (Table 1). In 77.7% (105/135) of the cases VAs were codominant, whereas in 19.3% (26/135) of the cases left VAs and in 3% (4/135) of the cases right VAs were dominant. In 4 cases right VAs and in 2 cases left VAs were becoming prominently thinner after giving the PICA branches (<0.5 mm). In 1 case left VA and in 3 cases right VAs were ending as PICA. There was no statistically significant difference between diameters of the vertebral arteries in males and females (*P* = 0.416, Mann-Whitney *U* Test).

**Figure 2:**
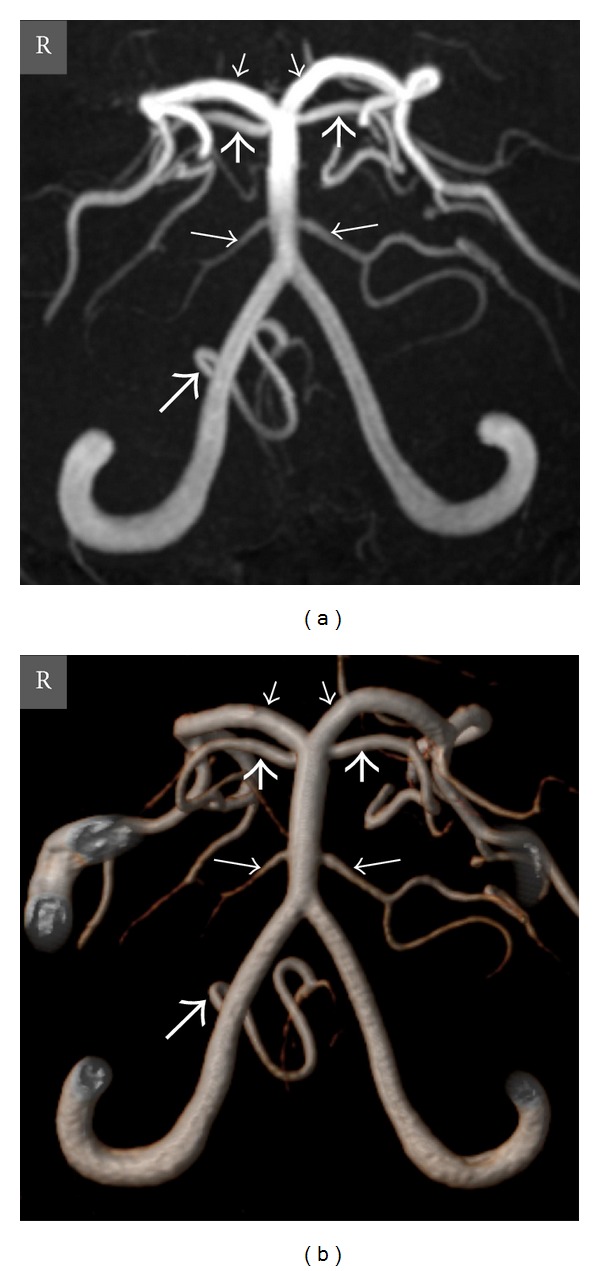
Oblique coronal view MIP (a) and VR (b) TOF MR angiography images show absence of the left PICA, and well-developed left AICA and right PICA in a 12-year-old girl. *Thick long arrow *= right PICA, *thin long arrows *= right and left AICAs, *thick short arrows *= right and left SCAs, *thin short arrows *= right and left PCAs, and *R* = right.

**Figure 3:**
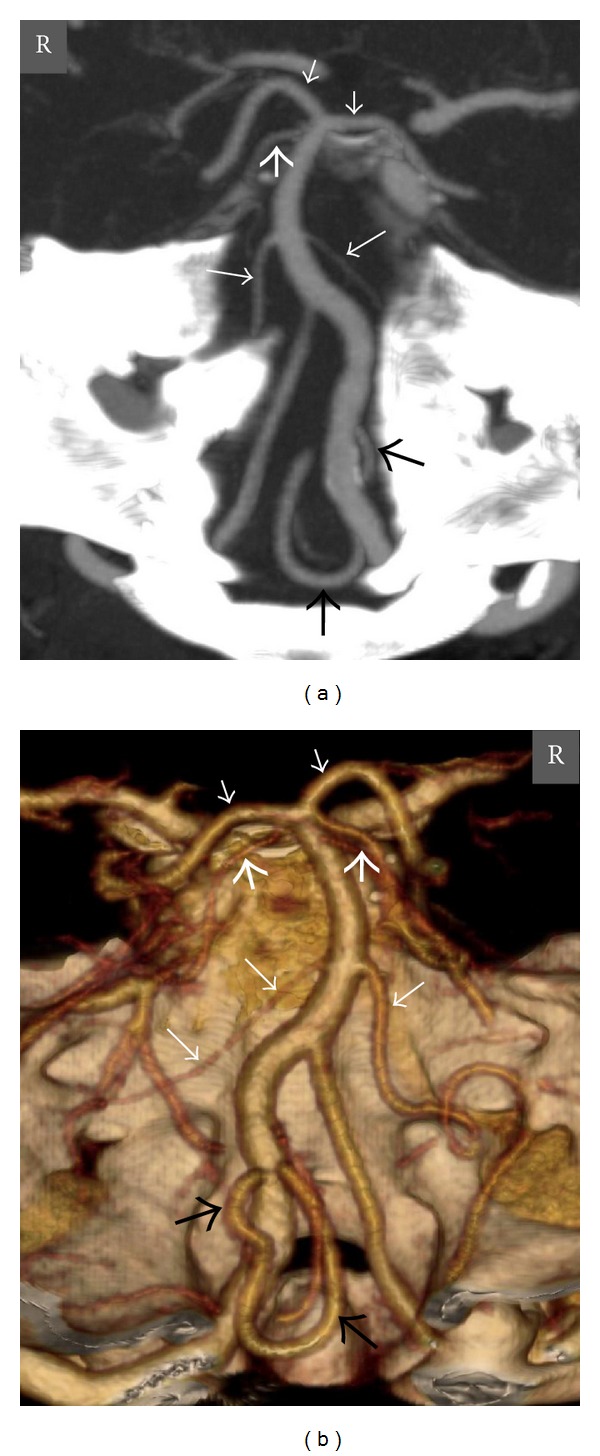
Oblique coronal view MIP (a) and posteroanterior view VR (b) computed tomography (CT) angiography images show absence of the right PICA and well-developed right AICA and left PICA in a 54-year-old man. The images also depict a dominant left vertebral artery. *Thick long arrow *= left PICA, *thin long arrows *= right and left AICAs, *thick short arrows *= right and left SCAs, *thin short arrows *= right and left PCAs, and *R* = right.

**Figure 4:**
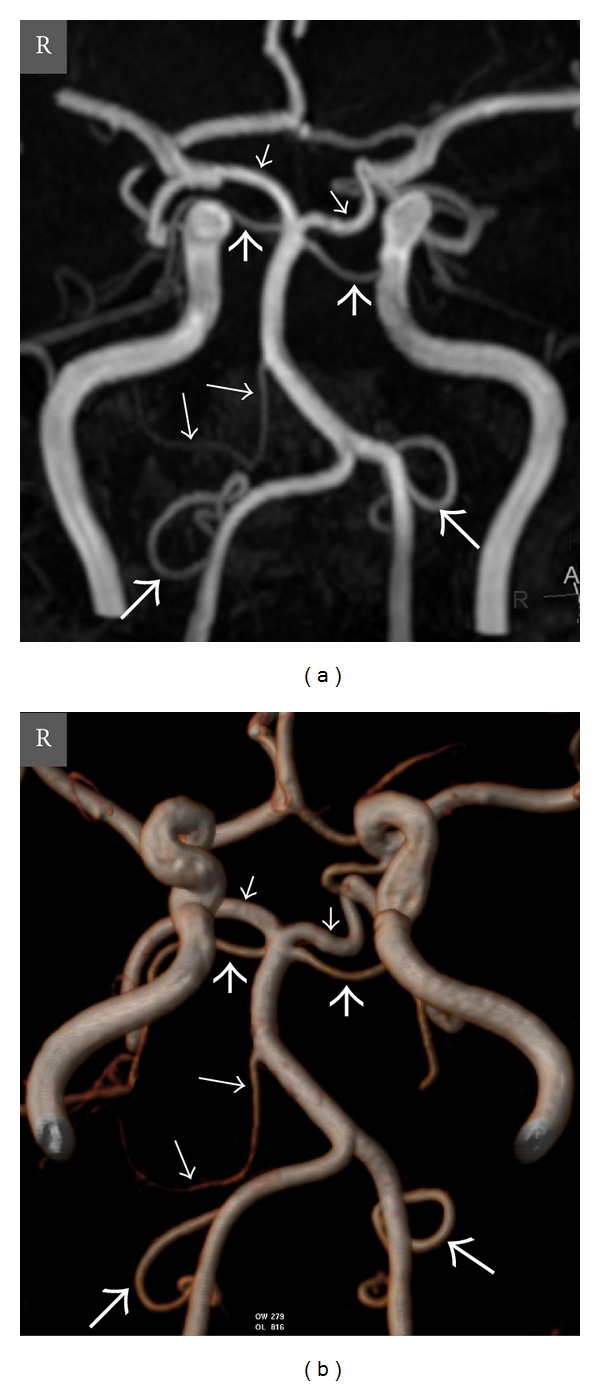
Oblique coronal view MIP (a) and VR (b) TOF MR angiography images show absence of the left AICA, well-developed left PICA, and moderately developed right PICA in a 48-year-old woman. *Thick long arrows *= right and left PICAs, *thin long arrow *= right AICA, *thick short arrows *= right and left SCAs, *thin short arrows *= right and left PCAs, and *R* = right.

**Figure 5:**
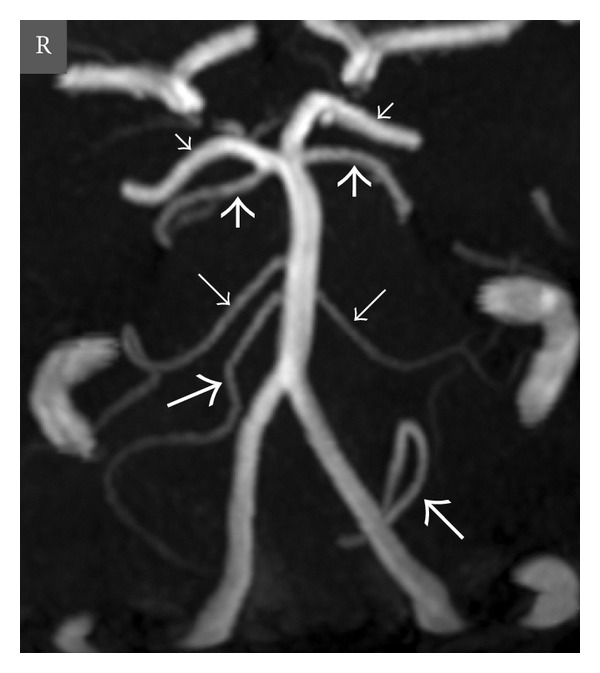
Oblique coronal view MIP TOF MR angiography image shows right PICA originating from the basilar artery in a 10-year-old girl. *Thick long arrows *= right and left PICAs, *thin long arrows *= right and left AICAs, *thick short arrows *= right and left SCAs, *thin short arrows *= right and left PCAs, and *R* = right.

 There was no PICA in 24.4% of the cases (33/135) on the right side and in 19.3% of the cases (26/135) on the left side. AICA was absent on the right side in 17.8% of the cases (24/135) and on the left side in 18.5% of the cases (25/135). There was well-developed right AICA in 33 cases without right PICA. There were 23 cases with well-developed and 3 cases with moderately developed left AICA in 26 cases without left PICA (Figures 2 and 3). There were 16 well-developed and 8 moderately developed right PICAs in 24 cases without right AICA. There were 21 well-developed and 4 moderately developed left PICAs in 25 cases without left AICA (Figure 4). There was no case with agenesis of both AICA and PICA on the same side. In cases with absent PICA on either side, there was statistically significant moderately or well-developed AICA on the same side and vice versa (*P* < 0.001, chi-square test). A significant negative correlation between the changes of the diameters of the ipsilateral PICAs and AICAs was revealed by using Spearman's rho analysis (Spearman's rho = −0.920 (right) and −0.925 (left), *P* < 0.001). There was no statistically significant difference between the ratios of the PICA and AICA absence or mean diameters of these vessels between gender groups or two imaging modalities (*P* > 0.05, chi-square test and Mann-Whitney *U* Test) (Table 1). In 4 cases (2 female, 2 male), right PICAs were originating from basilar artery (Figure 5). There was no left PICA originating from basilar artery in any of the cases. 

 There was no PICA on the right or left side in 4 cases. In these cases there were bilaterally well-developed AICAs. In 29 cases without right PICA (*n* = 33) and in 22 cases without left PICA (*n* = 26) there were moderately or well-developed contralateral PICAs. 

 In every case, there were one SCA and one PCA on each side. In 8 cases (5 male, 3 female) and in 6 cases (3 male, 3 female) P1 segments of the right PCAs and left PCAs were absent, respectively. In these 14 cases (10.4%) posterior cerebral arteries with fetal origin were demonstrated. In all of the cases, SCAs were originating from distal segment of the basilar artery; posterior cerebral arteries were distal branches of the basilar artery, except the ones with fetal origin. 

**Figure 6:**
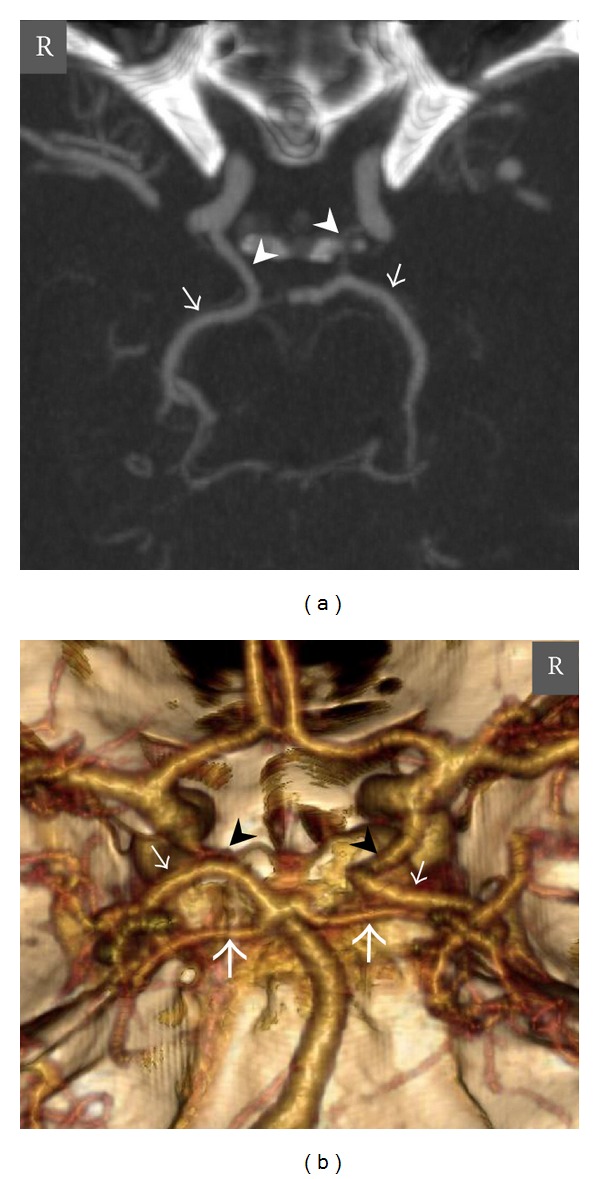
Axial MIP (a) and posteroanterior view VR (b) CT angiography images show a right PCA with fetal origin in a 42-year-old man. The P1 segment of the right PCA is agenetic, and there is a well-developed right PCoA that supplies the P2 and distal segments of the right PCA. The P1 segment of the left PCA is patent, and there is a thin calibrated left PCoA. *Thick short arrows *= right and left SCAs, *thin short arrows *= right and left PCAs, *arrowheads* = right and left PCoAs, and *R* = right.

 In 55 cases (30 male, 25 female) and in 56 cases (35 male, 21 female) there were no right PCoA and left PCoA, respectively. In 71 cases (47 male, 24 female) and in 74 cases (44 male, 30 female) there were fine calibrated right PCoAs and left PCoAs, respectively. In all of the cases (8 right, 6 left) that had PCAs with fetal origin, there were well-developed PCoAs (Figure 6). There was no statistically significant difference between genders according to the presence of the PCA with fetal origin in CT or MR angiographies. Additionally, there was no statistically significant difference according to the absence of the PCoAs (*P* > 0.05, chi-square test). 

 In our study group (*n* = 135) only 47 cases (34.8%) had well-known normal vertebrobasilar system anatomy. In the rest of the cases (65.2%), there was at least one anatomic variation. The most common variation was isolated agenesis of right PICA that was seen in 17.8% of the cases (24/135). The second one was isolated agenesis of left PICA that was seen in 11.1% of the cases (15/135). The variations and their frequencies that were encountered in our study group are presented in Table 2. There was at least one variation in 60.6% and 65.6% of the cases in CT and MR angiographies, respectively. There was no statistically significant difference between the frequencies of the variations in CT and MR angiography techniques (*P* = 0.643, chi-square test) or in gender groups (*P* = 0.282, chi-square test). 

 In our study group, we demonstrated fenestration of the basilar artery in 2 cases, fenestration of the left PCA in 1 case, dolichoectasia of the basilar artery in 14 cases, and vertebral/basilar artery indentation to the Bulbus/Pons in 16 cases. 

## 4. Discussion

Vertebrobasilar system supplies blood to the cerebellum and critical parts of the brainstem. As seen in any vasculature, variations of the major branches of the vertebrobasilar system are usually encountered. The most common variations reported in the literature are agenesis of AICA or PICA, AICA originating from PICA, PICA originating from internal carotid artery, persistence of a primitive communicating vessel (presegmental artery) between anterior and posterior circulation, and PICA originating from posterior meningeal artery [3]. 

 In our study group (*n* = 135) only in 47 cases (34.8%) there were well-known normal vertebrobasilar system anatomy. In the rest of the cases (65.2%), there was at least one anatomic variation. The most common variation was isolated agenesis of the right PICA (17.8%), and the second one was the isolated agenesis of the left PICA (11.1%) as reported previously in the literature. In 43.7% of the cases, one of the PICAs and in 36.3% of the cases one of the AICAs were absent. Territory of the agenetic vasculature was supplied by well-developed ipsilateral AICA or PICA and collateral branches coming from contralateral equivalent artery. We demonstrated no variation in any right or left SCAs. 

Arterial bypass procedure can be used in vertebrobasilar ischemia, skull base tumors, and complex and giant arteriovenous malformation related to posterior circulation. In this procedure PICAs, AICAs, SCAs, or PCAs are anastomosed end-to-end, end-to-side, or side-to-side to the contralateral equivalent arteries or to the extracranial arteries such as superficial temporal artery and the occipital artery to achieve the revascularization of the neural parenchyma [5, 6]. Although bypass procedures can reduce mortality and morbidity, the knowledge of the anatomic features (origin, diameter, and course) of the vasculature has an important role in preoperative planning of the appropriate branches and location for the anastomosis. Therefore, Shrontz et al. [5] obtained 27 unfixed human brains to 4–8 hours post mortem and injected polyester resin to the vertebrobasilar system to assess the anatomic features of the vertebrobasilar system, its main branches, and feasibility of microsurgery techniques. 

In this study, only 75% of the cases had PICA, and researchers reported that as a disadvantage for the microsurgery procedure. In our study group that was consisted of 135 cases, 33 cases (24.4%) without right PICA and 26 cases (19.3) without left PICA were demonstrated. Kawashima et al. [6] evaluated the anatomic features of the vertebrobasilar system and its main branches in 22 adult cadaveric specimens in their research focused on revascularization of the cerebral parenchyma. They measured the mean diameters of the PICA, AICA, SCA and PCA as we did in our study and reported as 1.84 mm, 1.34 mm, 1.67 mm, and 2.13 mm, respectively. The values in our study were parallel to those results. But, in that study, the anatomic variations were not mentioned.

 Infarcts of the vertebrobasilar circulation are responsible for 12–27% of all strokes in hospitalized patients, and a significant number of these are due to basilar artery disease [13, 14]. The underlying cause, the cerebrovascular anatomical variations, interarterial anastomoses, and the hemodynamic situation are the major factors that determine the location and extent of the infarcts. The underlying cause is primarily classified as embolism or thrombosis, and location is also mentioned [15]. 

 While interpreting the cause and the outcome of the ischemic events, the variations of the vertebrobasilar circulation that are frequently encountered should be kept in mind. In particular the variations in origins, diameters, irrigation areas, and anastomoses of the arteries may lead to different clinical outcomes in different cases. In our study group, there was at least one anatomic variation in 63% of the cases. The most common variation was isolated agenesis of one of the PICAs. In the vast majority of these cases, the blood supply of the agenetic PICA territory was coming from the ipsilateral well-developed AICA or from the contralateral PICA by interarterial anastomosis. In these cases, an occlusive process effecting AICA also leads to ischemia and symptoms due to territory of the PICA. The knowledge of the common variations of the vertebrobasilar circulation may be important in interpreting clinical symptoms and the anatomic features of the vasculature in MRI, CT, and MR angiographies. 

 Although DSA is the gold standard method in assessment of the vascular system, its invasive nature and requirement of ionizing radiation are disadvantages of this technique. With recent advances in CT and MR angiography techniques, these methods have become more commonly used in demonstrating variations of the vascular structures [8–12]. In particular CT angiography can readily depict vascular map of any part of the body in a few seconds. The major disadvantages of this technique are requirement for ionizing radiation and iodinated contrast media that have potential nephrotoxic effects [8–10]. On the other hand, MR angiography can be performed without contrast medium and does not require ionizing radiation. In previous studies, TOF MR angiography was reported to be more sensitive than CT angiography in demonstrating intracerebral arteries especially in equipments with high magnetic field [11]. In our study we did not determine any statistical difference between these two modalities. Some of the potential disadvantages of the MR angiography are relatively longer imaging time, susceptibility to motion artifacts, contraindication for some medical equipments, or foreign substances that are not compatible with MR. Additionally, the patients with claustrophobia may not tolerate this technique. 

Our study had several limitations. First of all, it is a retrospective study that is consisted of only CT or MR angiographies. Some of the branches of the vertebrobasilar circulation with fine calibration might not be visualized by CT or MR angiographies. DSA confirmation or microsurgical dissections were not performed in these cases. In particular in patients with beam hardening artifacts or motion artifacts, fine calibrated vessels might not be visualized properly. Second, in our study, patients were evaluated by either CT angiography or MR angiography. Although there was no statistically significant difference between these modalities demonstrating variations of the vertebrobasilar circulation, for appropriate comparison, both of these modalities should be performed for each case. Third, while evaluating the variations, we focused on the origins of the vessels. But especially in cases with variations, the branching pattern and distribution of the moderately or well-developed vessels supplying the territory of the agenetic vessel were not evaluated. Although prospective studies with healthy cases performed by using CT, MR angiography, and DSA can avoid these limitations, the requirement for ionizing radiation and contrast medium are the major handicaps. 

## 5. Conclusion

The anatomic features of the branches of the vertebrobasilar circulation may be different from well-known normal anatomy. In patients with acute cerebral vascular accidents or pathologies of vertebrobasilar circulation such as aneurysm or atherosclerotic disease that are candidate for interventional or microsurgical treatment, the unfamiliarity with the common variations of the vertebrobasilar circulation may cause misinterpretation of the causes and results and lead to wrong management. CT and MR angiographies allow a precise and detailed evaluation of this system that is supplying critical zones such as brainstem, cerebellum, and important cerebral parts.

## Figures and Tables

**Table 1:** Diameters of the branches of vertebrobasilar circulation.

	Artery (mean diameter ± SD mm)				
	VA	PICA	AICA	SCA	PCA
Right	2.95 ± 0.47	1.67 ± 0.45	1.24 ± 0.38	1.59 ± 0.39	2.56 ± 0.43
Left	3.23 ± 0.57	1.64 ± 0.37	1.28 ± 0.42	1.52 ± 0.31	2.43 ± 0.34
Total	3.08 ± 0.53	1.66 ± 0.53	1.26 ± 0.43	1.56 ± 0.44	2.52 ± 0.36

VA: vertebral artery, PICA: posterior inferior cerebellar artery, AICA: anterior inferior cerebellar artery, SCA: superior cerebellar artery, and PCA: posterior cerebral artery.

**Table 2:** Variations of the vertebrobasilar circulation.

Types of variation	CTA	MRA	Total
Normal anatomy	26	21	47
Agenesis of right PICA	19	5	24
Agenesis of left PICA	9	6	15
Agenesis of right AICA and left PICA	2	7	9
Agenesis of right and left AICA	2	7	9
Agenesis of left AICA	2	7	9
Agenesis of right AICA	4	2	6
Agenesis of right PICA and left AICA	3	2	5
Agenesis of right and left PICA	2	2	4
Right PICA originating from basilar artery	0	3	3
Right PICA originating from basilar artery and agenesis of left AICA	0	1	1
Isolated fetal origin of the PCA*	2	1	3

Total	71	64	135

CTA: computed tomography angiography, MRA: magnetic resonance angiography, PICA: posterior inferior cerebellar artery, AICA: anterior inferior cerebellar artery, and PCA: posterior cerebral artery.

*The other 11 cases of fetal origin of the PCA were accompanied to agenesis of PICA and/or AICA.
